# The Reaction to Social Stress in Social Phobia: Discordance between Physiological and Subjective Parameters

**DOI:** 10.1371/journal.pone.0105670

**Published:** 2014-08-25

**Authors:** Elisabeth Klumbies, David Braeuer, Juergen Hoyer, Clemens Kirschbaum

**Affiliations:** 1 Institute of Biopsychology, Technische Universitaet Dresden, Dresden, Germany; 2 Institute of Clinical Psychology and Psychotherapy, Technische Universitaet Dresden, Dresden, Germany; Max Planck Institute for Human Cognitive and Brain Sciences, Germany

## Abstract

**Background:**

Research on the biopsychological background of social phobia (SP) is scarce and inconsistent. We investigated endocrine and autonomic markers along with subjective responses to a standardized stress situation (Trier Social Stress Test, TSST) in SP patients and healthy controls (HC).

**Methods:**

We examined 88 patients with the primary diagnosis of SP as well as 78 age and sex comparable HCs with the TSST. Blood and saliva samples were obtained before and after the TSST for the assessment of salivary cortisol, plasma cortisol, salivary alpha-amylase (sAA), and prolactin. Heart rate (HR) and heart rate variability (HRV) were recorded continuously. Scalp-near hair samples were collected for the assessment of long-term cortisol secretion. The self-reported stress response was measured with different state and trait scales.

**Results:**

While self-reported anxiety was elevated in SP before, during, immediately after, and one week after the TSST, no significant differences in biological stress responses were observed between SP and HC. There was a trend for SP to show higher baseline stress markers. Also long-term cortisol deposition in hair remained unaltered.

**Conclusions:**

Our results suggest that the excessive self-reported stress in SP is not reflected by a respective biological stress response. Patients with SP apparently show neither an extreme form of focused fear reactivity nor excessive defensive impairment.

## Introduction

Social phobia (SP) is a common mental disorder with lifetime prevalence rates in Western cultures between 7 and 12% [1], [2]. It is characterized by persistent and excessive fear of one or more social or performance situations, in which the person is exposed to other people or to possible scrutiny by others. The individual fears refer to acting in a way that will cause humiliation or embarrassment [3]. In spite the fact that SP is a common mental disorder, it is not clear whether the high self-reported anxiety of SP patients in social situations corresponds to an abnormal physiological reaction pattern [4]. Clarification of the physiological basics of SP could contribute to new therapy approaches and better indicative decisions [5].

Social situations cause stress for the SP patient. Subjectively, being stressed means experiencing feelings of anxiety and threat, as well as corresponding cognitions. Biologically acute stress activates the two major stress pathways, i.e., the hypothalamic-pituitary-adrenal (HPA) axis and the autonomic nervous system (ANS) [6]. In addition, a variety of other biological indicators responds to acute psychosocial stress [7].

Equivocal results have been published on the biological stress response of SP patients. A number of studies reported no significantly higher reactivity in SP (as compared to HCs) in response to a social stressor (a) for salivary cortisol [4], [8]; (b) for plasma cortisol [9], (c) for sAA [4], and (d) for heart rate [10]–[13]. For HRV, no study reported higher reactivity in SP.

Significant differences between SPs and HCs in response to a social stressor reported (a) for salivary cortisol [10], [14]–[16]; (b) for plasma cortisol [17]; (c) for sAA: no study; (d) for HR [4], [18], [19], and (e) for HRV [20]”.

To our best knowledge, no previous study examined the prolactin stress response of SP patients in contrast to HCs. For healthy individuals, several studies found an increased level in response to psychosocial stress [21]–[23] while one study did not find any stress-induced increases [24].

Furthermore, no previous study investigated long-term cortisol secretion of SP patients, as it has become measurable though new assessment tools in human hair [25]. However, short-term basal levels for some hours (urine) or some minutes (cortisol in plasma and saliva) in SP patients were found to be comparable to HCs in all studies [26]–[28]. There are several factors which could contribute to the inconsistencies of previous research concerning the stress response of SP patients: Firstly, very different and mostly non-standardized stressors were used (e.g., interaction vs. performance situations), thus making it harder to conduct a comparison between several studies. Secondly, some stressors were possibly not sufficiently effective to induce anxiety and/or significant biological responses [8], [9]. Thirdly, differences in sample characteristics like age or comorbidity status might have caused different results: From studies of SP children [4], [16], [20] no final conclusions can be drawn to SP adults, as age influences baseline values and/or reactivity to the TSST of several physiological parameters [29]–[31]. Furthermore, differences in patients' comorbidity status may have influenced the results of previous studies [32]. Fourthly, biological differences may exist in SP patient subgroups. Furlan et al. [14] found a significantly higher number of SP non-responders along with a greater decrease in salivary cortisol throughout the study than in HC non-responders. SP cortisol responders, however, showed a greater stress-induced increase than the HC responders. Fifthly, many studies lacked adequate statistical power to detect smaller effects. Lastly, most studies investigated only one or two biological parameters (mostly HR or salivary cortisol).

To overcome some of the mentioned limitations, the present study applied a well-established standardized social stress test [22], to reliably elicit a moderate and comparable stress response in both patients and HCs. Secondly, we attempted to evaluate a more comprehensive pattern of acute stress responses in SP by the measurement of several endocrine and autonomic parameters in a larger group of SP patients using a most potent laboratory stress protocol. Salivary and plasma cortisol, plasma prolactin, salivary alpha amylase, HR, and HRV, along with several self-report rating scales, served as biological and psychological stress parameters. Thus, we investigated the stress response towards a standardized social stress test on a comprehensive level, and were able to measure autonomic, endocrine and subjective responses at one measurement point, in a sufficiently large sample.

We expected a higher subjective HPA axis (salivary cortisol, plasma cortisol), autonomic (HR, HRV, sAA), and prolactin stress response of SP patients compared to HCs. Furthermore, we expected higher self-reported anxiety and stress symptoms within the patient group. Additionally, as there were no specific, directed hypotheses for basal physiological parameters as well as hair cortisol concentration (a measure of long-term cortisol production) we statistically explored potential differences between SP and HC for these variables.

## Methods

### Participants

Eighty-eight SP patients (44 females) and n = 78 HCs (37 females) comparable in age and sex participated in this study. The patients were recruited in the outpatient clinic of the Institute of Clinical Psychology and Psychotherapy and at the Carl Gustav Carus University Hospital of the TU Dresden (Germany). We conducted the Munich-Composite International Diagnostic Interview (DIA-X/M-CIDI) [33] to confirm DSM-IV diagnoses for social phobia and comorbid mental disorders. Patients were included if they met the DSM-IV criteria for social phobia as a primary diagnosis and reached a score of ≥30 in the Liebowitz Social Anxiety Scale (LSAS, German version) [34]. They were excluded if they presented any comorbid substance-related disorder, psychotic disorder, personality disorder (except for avoidant, dependant or obsessive-compulsive disorder), or organic mental disorder. At the time of the study, none of the patients was in psychotherapeutic treatment. HCs were recruited by advertisement in the local newspapers and flyers. They were included if the stem questions of the DIA-X/M-CIDI indicated no lifetime psychiatric disorder [35] and if their LSAS score was <30. Exclusion criteria for all subjects were as follows: any physical condition or intake of medication that influences the HPA axis, smoking more than 10 cigarettes per day, pregnancy, or breastfeeding. All women were tested in the luteal phase of their menstrual cycle as indicated by self-report.

A total of 98 patients and 80 healthy controls were recruited for participation, from whom 12 individuals had to be excluded due to various reasons (n = 2 already knew the TSST from a former study; n = 1 refused the TSST, n = 2 with blood-injection-injury phobia; n = 2 with substance dependence; and n = 5 took HPA axis relevant medication [n = 1 cortison-containing nasal spray, n = 4 antidepressants]). Finally, n = 88 (44 females; age = 29.69, SD = 9.55) patients with social phobia and n = 78 (37 females; age = 30.22, SD = 9.96) remained for statistical analyses. The two groups did not differ significantly regarding age, gender, intake of hormonal contraceptives, BMI, or smoking. However, SPs took significantly more medication (antihistamines, thyroid agents, proton pump inhibitor, analgesic, nutritional supplements, homeopathy) than HCs (*p* = .045; see table 1).

**Table 1:** Group characteristics of patients with social phobia (SP) and healthy controls (HC).

*N* = 166		SP (n = 88)		HC (n = 78)		Test	*p*	df
**Age (years)**	*M* (*SD*)	29.69	(9.55)	30.22	(9.96)	*T* = −0.35	.729	164
**Gender (female)**	*n* (%)	44	(50.0)	37	(47.0)	χ^2^ = 0.11	.742	1
Orale contraceptives	*n* (%)	22	(22.7)	16	(20.5)	χ^2^ = 0.12	.730	1
Menopause	*n* (%)	4	(4.5)	2	(2.6)	*Fisher's*	.685	1
**BMI (kg/m** 2 **)**	M (SD)	22.93^1^	(3.39)	23.13	(2.70)	*T* = −0.41	.683	163
**Smoking (yes)**	*n* (%)	15^1^	(17.2)	8	(10.3)	χ^2^ = 1.67	.196	1
**Medication (yes)**	*n* (%)	11	(12.5)	3	(3.8)	χ^2^ = 4.01	**.045** *	1
Antihistamine	*n* (%)	1	(1.1)	0	(0.0)			
Thyroid agents	*n* (%)	3	(3.4)	0	(0.0)			
Proton pump inhibitor	*n* (%)	1	(1.1)	0	(0.0)			
Nutritional supplements	*n* (%)	2	(2.3)	3	(3.8)			
Homeopathy	*n* (%)	1	(1.1)	0	(0.0)			
Analgesic	*n* (%)	3	(3.4)	0	(0.0)			
**Social Phobia**								
LSAS (total score)	*M* (*SD*)	68.16	(18.57)	10.10	(7.34)	*T* = 27.57	**<.001** ***	134.95
SPAI	*M* (*SD*)	3.88	(0.83)	0.98	(0.66)	*T* = 24.48	**<.001** ***	163
**Psychological variables**								
TPQ Harm Avoidance	*M* (*SD*)	23.27	(5.51)	9.40	(4.81)	*T* = 16.73	**<.001** ***	154
TPQ Novelty Seeking	*M* (*SD*)	13.58	(5.50)	16.35	(4.83)	*T* = −3.34	**.001** ***	154
TPQ Reward Dependance	*M* (*SD*)	17.35	(4.39)	17.41	(3.72)	*T* = −0.10	.922	154
Depression (BDI)^1^	*M* (*SD*)	13.16	(7.65)	2.22	(2.91)	*T* = 12.43	**<.001** ***	114.49
Anxiety Sensitivity (ASI)^2^	*M* (*SD*)	42.24	(13.53)	27.99	(8.51)	*T* = 7.48	**<.001** ***	113.09
**Comorbidity (lifetime prevalence)**	*n* (%)	53	(60.2)					
Alcohol abuse	*n* (%)	5	(5.7)					
Cannabinoid/sedativa abuse	*n* (%)	2	(2.3)					
Depressive d	*n* (%)	19	(21.6)					
Dysthymic d	*n* (%)	16	(18.2)					
Agoraphobia	*n* (%)	3	(3.4)					
Panic d	*n* (%)	4	(4.5)					
Specific phobia	*n* (%)	6	(6.8)					
Generalized anxiety d	*n* (%)	6	(6.8)					
Obsessive compulsive d	*n* (%)	1	(1.1)					
Adjustment d	*n* (%)	2	(2.3)					
Somatoform d	*n* (%)	9	(10.2)					
Avoidant personalty d	*n* (%)	10	(11.4)					

*Notes*: *T* = T-Test, *p* = p-value; df = degrees of freedom, *M* = mean, *SD* = standard deviation; BMI  =  body mass index; LSAS  =  Liebowitz Social Anxiety Scale; SPAI  =  Social Phobia Anxiety Inventory; TPQ  =  Tridimensional Personality Questionnaire; BDI  =  Beck Depression Inventory; d = disorder.

1 N = 165 because of one missing value.

2 N = 143 because of missing values.

* = *p*<.05,

** = *p*<0.01,

*** = *p*<.001.

Written informed consent was obtained from all subjects prior to their inclusion in the study and the study protocol was approved by the local ethics committee (Ethikkommission der Technischen Universitaet Dresden, Germany). The volunteers were remunerated with 25 Euro.

### Procedure

The test comprised three phases: A 60-min rest period, starting after the explanation of the test procedure and the insertion of the indwelling catheter, was followed by a 15-min stressor and a 60-min post-stressor period. All participants underwent the TSST [22], a standardized public speaking task involving a short preparation period (5 minutes), a public speaking task (5 min) followed by a mental arithmetic task (5 min) in front of a committee. Blood and saliva samples were obtained 45 and 1 min before, and 1, 10, 20, 30, 45 and 60 min after the stressor). HR and HRV were continuously recorded. Hair samples were taken at the end of the post-stressor period. Furthermore, we assessed subjective appraisal and anxiety by questionnaires (see psychological parameters).

### Physiological parameters

#### Hair cortisol

Scalp-near hair strands were taken from a posterior vertex position from participants with a total hair length of at least 3 cm. The scalp-near hair segment (3 cm) was used for the analysis of cortisol concentration representing hair growth over a period of approximately 3 months [36]. Hair steroid extraction and cortisol determination were performed following the protocol published in detail elsewhere [37].

#### Blood parameters: plasma cortisol and prolactin

For the assessment of plasma cortisol and prolactin levels, a catheter was inserted 60 min prior to the TSST into the antecubital vein. Eight blood samples were drawn using EDTA-coated monovettes (Sarstedt, Nümbrecht, Germany) and stored at −80°C until biochemical deterimination. Plasma cortisol and prolactin levels were analyzed using commercial ELISAs (IBL International, Hamburg, Germany). Intra- and interassay variances were below 6% for plasma cortisol and prolactin.

#### Salivary parameters: cortisol and salivary alpha-amylase

Eight saliva samples were collected with Salivettes (Sarstedt, Nümbrecht, Germany) for later determination of salivary free cortisol and sAA levels and stored at −20°C until biochemical analysis. For analysis, saliva samples were centrifuged at 3000 rpm for 3 minutes after thawing. Salivary free cortisol was measured using a chemiluminescence immunoassay (CLIA; IBL International, Hamburg, Germany). For sAA analysis, we applied a quantitative enzyme-kinetic method [38]. Intra- and interassay coefficients of variation were below 4% for salivary cortisol and sAA.

#### Heart rate and heart rate variability

HR and HRV as additional markers of autonomic activity were measured and stored continuously employing Polar S810i cardiac monitors (Polar Electro Ltd., Kempele, Finland) using the RR-date modus. The time solution of 1 ms in the RR-date modus, allows the analysis of HRV. Before analyzing both parameters, the raw interbeat intervals were preprocessed for artifacts using the Polar Precision Software. The subsequent analyses were performed with HRV Analysis Software (Biomedical Signal Analysis Group, University of Kuopio, Kuopio, Finland). For analysis, the following seven time intervals of 2-min duration were chosen: two intervals from the 60 min acclimation period (−30, −5 min prior to the TSST), two under stress (TSST speech, TSST math) and three at recovery (7, 17, 27 min after cessation of the TSST). These intervals were chosen in order to not interfere with blood collection time points. For these intervals mean HR (in beats per minute, BPM) and the root mean square of successive differences (RMSSD) in milliseconds from time domain analysis were calculated. RMSSD is defined as the variability between two heart beats and is, as it stands, a marker of parasympathetic response, scarcely explored in SP by date. The potential influence of respiration was neglected following Penttila et al. [39], reporting that RMSSD is not significantly affected by breathing rate, despite high correlations to power spectral measures of sinus arrhythmia. All measures (e.g. length of time intervals etc.) were in accordance to the guidelines of the Task Force of the European Society of Cardiology and the North American Society of Pacing and Electrophysiology [40].

### Psychological parameters

To assess social phobia, the total score of the clinician-administered Liebowitz Social Anxiety Scale (LSAS, German version) [34], and the Social Anxiety subscale of the Social Phobia and Anxiety Inventory (SPAI, German version) [41] were employed. To assess depressive symptoms, the Beck Depression Inventory was used (BDI-II, German version) [42]. Temperament was assessed using Cloninger's Tridimensional Personality Questionnaire (TPQ, German version) [43], which contains the scales “Novelty Seeking”, “Harm Avoidance”, and “Reward Dependence”. Anxiety sensitivity was measured with the Anxiety Sensitivity Index (ASI) [44].

We assessed anticipatory cognitive appraisal processes after the 3 minutes preparation time during the TSST with the Primary Appraisal Secondary Appraisal questionnaire (PASA) [45]. Changes in mood and state anxiety were assessed immediately before and after the TSST with the three scales “Good Mood versus Bad Mood”, “Calmness versus restlessness”, and “Alertness versus Tiredness” of the German Multidimensional Mental-State Questionnaire (MDBF) [46] and the state version of the State-Trait Anxiety Inventory (Stai-S) [47], respectively. We measured subjective perceptions of the TSST with eight visual analogue scales (VAS; range 0–100) [45] regarding how novel, unpredictable, challenging, uncontrollable, threatening, and stressful the TSST was perceived, as well as participants' ego-involvement and satisfaction with their performance. Negative post event processing was assessed one week after the TSST via telephone with the Post Event Processing Questionnaire (PEPQ, German version) [48].

### Statistical analyses

All statistical analyses were performed with IBM SPSS Statistics 19. If there were no more than three out of eight physiological values of a participant missing (1.28% of blood and salivary data respectively; 1.04% of HR/HRV data), they were replaced with linear transformation (time points 5 to 7) or the expectation maximization algorithm (time points 1-4 and 8) in SPSS [49]. If there were four or more values missing, the participant was excluded from further analysis. Outliers with z-scores more than +/− 3 standard deviations (depending on the physiological parameter 0–5 participants), and missing values due to technical problems were excluded from statistical analyses. This resulted in varying degrees of freedom for the dependent variables. Prolactin (46 SP/34 HC) and hair cortisol (33 SP/20 HC) were analyzed in subsamples.

We tested data for normal distribution and homogeneity of variance using Kolmogorov–Smirnov and Levene's tests, as well as for skewness, before statistical procedures were applied. These analyses revealed significant deviations of some (of the sequence 8) plasma cortisol, prolactin, salivary free cortisol, sAA, HR, and HRV values. Therefore, they were all square root transformed.

Differences in plasma cortisol, salivary free cortisol, prolactin, and sAA to the TSST were computed by separate two-way analyses of variance (a) for the rest period with group (SP/HC) as the between-subject factor and time (−45 to −1 min) as the repeated measures factor, as well as (b) for the stress period with group (SP/HC) as the between subject factor and time (−1 to 60 min) as the repeated measures factor. Similarly, differences in HR and HRV were computed by separate two-way analyses of variance (a) for the rest period with group (SP/HC) as the between-subjects factor and time (−30 to −5 min) as the repeated measures factor, and (b) for the stress period with group (SP/HC) as the between-subjects factor and time (−5 to 27 min) as the repeated measures factor. Greenhouse-Geisser and Bonferroni corrections were applied where appropriate. Mean differences were calculated with Student's t-tests. For significant results, we report partial eta squared (η^2^) as a measure for effect size. For categorical data, Chi-Square tests were used. The significance level was alpha  = 0.05 for all analyses (two-tailed).

## Results

### Biological stress responses to the TSST

**Figure 1:**
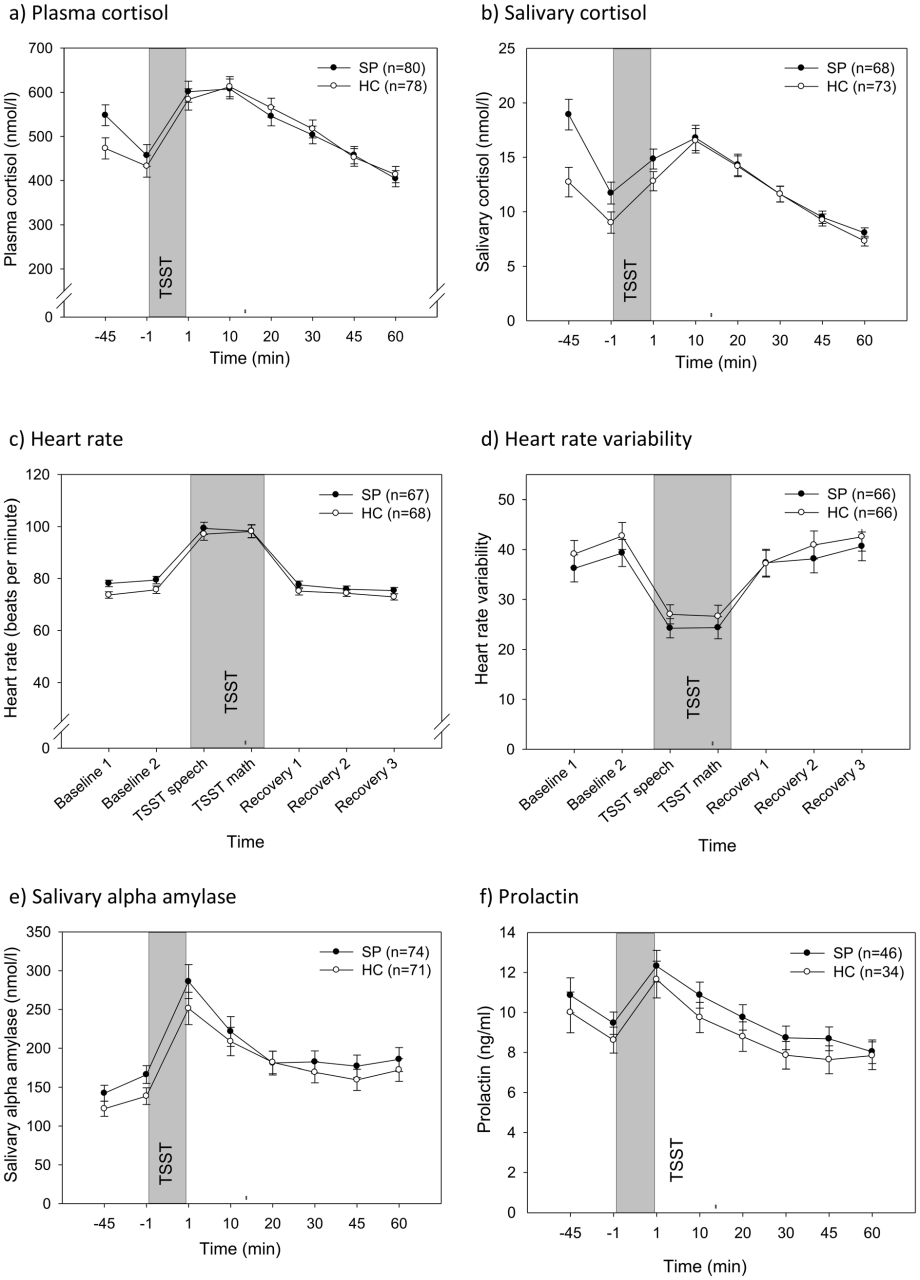
Means (±SEM) of physiological responses to the Trier Social Stress Test (TSST) in social phobia patients (SP) and healthy controls (HC).

Figure 1a - f summarizes the physiological responses to the TSST in SPs and HCs, respectively. Regarding the rest phase (t1-t2), ANOVAS with ‘group’ (SP/HC) as the between- and ‘time’ as the within-factor showed a significant main effect for time for all physiological parameters (all F>6.0, all *p*<.016, all η^2^>.043), indicating a decrease in salivary cortisol, plasma cortisol, and prolactin levels, in addition to an increase in HR, HRV, and sAA from the first to the second time point. For the stress phase, ANOVAS with group (SP/HC) as the between- and time as the within-factor showed substantial main effects for time for all physiological parameters (all F>48.0, all *p*<.001, all *η^2^*>.381). The average TSST-induced increase of salivary and plasma cortisol, HR, and sAA (with a respective decrease for HRV) from baseline levels (t2) was between 26.64% (HR) and 76.24% (sAA) in all subjects. Further on, ANCOVAs evaluated the effect of the covariate sex on the dependent physiological variables. In no case did inclusion of this covariate alter the results presented (data on request).

### Hypothalamic-pituitary adrenal axis responses to the TSST

#### Salivary cortisol responses

Mean absolute levels of salivary cortisol for SPs and HCs are presented in Figure 1a. Concerning the rest phase, the results of the 2×2 Group (SP/HC)×Time (−45, −1 min) ANOVA revealed a significant main effect for group (F(1,139) = 3.19, *p* = .005, *η^2^* = 0.056), which was modulated by a time×group interaction (F(1,139) = 6.80, *p* = .010, *η^2^* = 0.046). Post hoc comparisons of the two groups revealed significantly higher salivary cortisol levels of SP patients relative to HCs at both time points during the rest phase (all *p*<.035).

**Figure 2:**
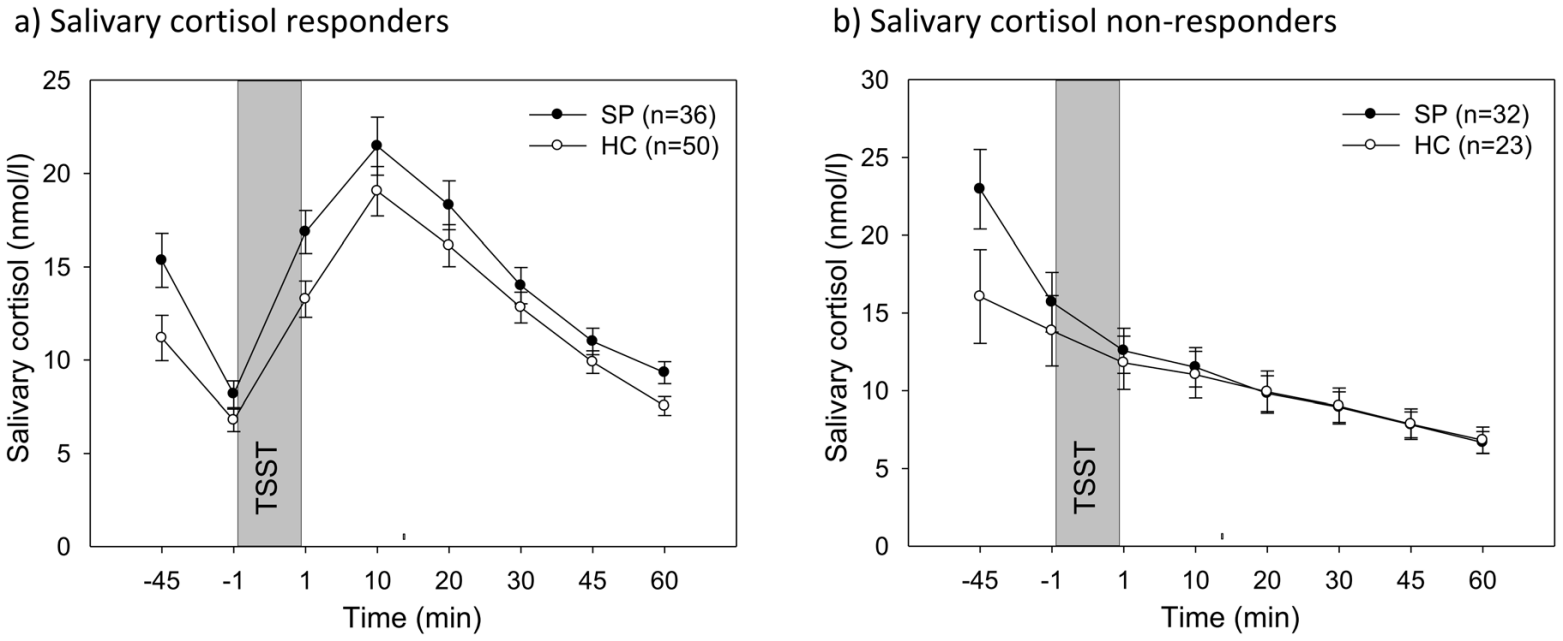
Means (±SEM) of salivary cortisol responders and non-responders to the TSST for social phobia patients (SP) and healthy controls (HC).

For the stress phase, a 2×7 group (SP/HC)×time (−1 to 60 min) ANOVA with repeated measures yielded no significant main effect for group (F(1,139) = 0.48, *p* = .492), but a significant time×group interaction (F(1.9,270.0) = 3.52, *p* = .032, *η^2^* = 0.025). This interaction, however, was primarily due to the over-representation of salivary cortisol non-responders with their increased baseline profile in the patient group: 32 SPs (47.1%), but only 23 HCs (31.5%) were classified as non-responders. This distribution of responders/non-responders between both groups yielded a trend towards significance (χ^2^(1) = 3.58, *p* = .059). In order to control for this effect, we computed separate analyses for responders and non-responders, respectively (see Figure 2a/b). As a result, the seemingly significant interaction between SPs and HCs regarding the stress response disappeared (all F<2.33, all *p*>.131). The significant interaction in the acclimation phase remained for the responders (F(1,84) = 4.92, *p* = .029, *η^2^* = 0.055), and as trend for the non-responders (F(1,53) = 3.93, *p* = .053, *η^2^* = 0.069). There were no significant differences in demographic variables between the four groups except for intake of oral contraceptives (χ^2^(3) = 9.04, *p* = .027) with fewer subjects taking oral contraceptive in both responder groups.

#### Plasma cortisol responses

Figure 1b shows the mean absolute levels of plasma cortisol for SPs and HCs for each time point. With respect to the rest phase, the results of the 2×2 Group (SP/HC)×Time (−45, −1 min) ANOVA indicated a trend towards a significant main effect for Group (F(1,156) = 3.19, *p* = .076, *η^2^* = 0.02), and a significant Time×Group interaction (F(1,156) = 4.14, *p* = .044, *η^2^* = 0.026). Post hoc analyses revealed significantly higher plasma cortisol levels of SP patients at the first (−45 min: *p* = .017), but not at the second time point (−1 min: *p* = .304).

For the stress phase, a 2×7 Group (SP/HC)×Time (−1 to 60 min) ANOVA with repeated measures on the Time factor yielded no significant main effect for Group (F(1,156)<1), but a tendency towards a significant Time×Group interaction (F(2.4,373.3) = 2.38, *p* = .084). These findings suggest that the plasma cortisol response to the TSST was similar in both groups.

### Autonomic nervous system responses to the TSST

#### Heart rate responses

Figure 1c shows the mean absolute levels of heart rate for SP and HC in each experimental phase. For the rest phase, a 2×2 Group (SP/HC)×Time (−30, −5 min) ANOVA with Time as repeated measures factor yielded a significant main effect for Group (F(1,133) = 5.01, *p* = .027, *η^2^* = 0.036), but no significant Time×Group interaction (F(1,133) = 0.95, *p* = .331). Among both groups, the SPs had higher HR levels during the acclimation phase (−30 min: *p* = .012*; −5 min: *p* = .086(*)).

For the stress phase, a 2×6 Group (SP/HC)×Time (−5 to 27 min) ANOVA with repeated measures on the Time factor yielded no significant main effect for Group, and no significant Time×Group interaction (all F<1) suggesting similar HR responses to the TSST in both groups.

#### Heart rate variability responses

Mean absolute levels of HRV for both groups are presented in Figure 1d. There were no significant effects for Group and Time×Group; neither in the rest phase nor in the stress phase (all F<1.2; all *p*>.28).

#### Alpha-amylase responses

Mean absolute levels of sAA for the SP and HC group are presented in Figure 1e. For the rest phase, there was a tendency towards a significant Group effect (F(1,141) = 2.70, *p* = .103), but no significant effect for Group×Time (F(1,141)<1). In the stress phase, there were no significant effects for Group and Time×Group (all F<1.2; all *p*>.343).

#### Prolactin responses to the TSST

Both groups showed similar prolactin response patterns both in both the rest and stress phases (see figure 1f); there were no significant effects for Group and Time×Group (all F<1).

#### Basal levels of cortisol in hair

**Figure 3:**
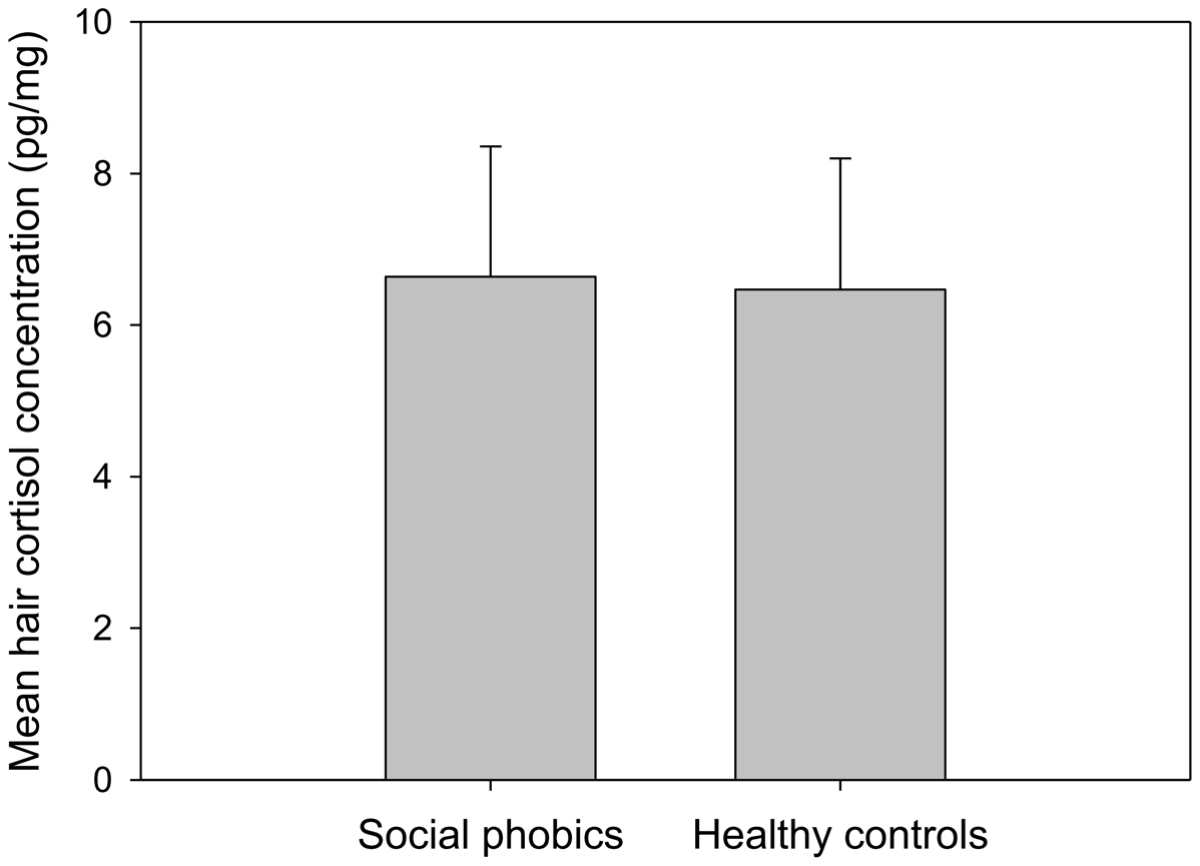
Mean (±SEM) hair cortisol concentrations of patients with social phobia (SP) and healthy controls (HC).

Figure 3 illustrates mean hair cortisol concentrations for SP patients and HCs. There were no significant differences between both groups regarding hair cortisol concentrations (t = 0.10; *p* = .918).

### Subjective stress parameters

Table 2 shows the mean levels of self-reported anxiety and mood ratings for both groups. SP patients and HCs differed significantly regarding their primary and secondary appraisal: SP patients interpreted the TSST more as a threat than as a challenge with less possibilities to cope with the stressful situation (all *p*<.001). Immediately after the TSST, SP patients evaluated the stressful situation using the VAS as more unpredictable, stressful, uncontrollable, threatening, and were less content with their performance (all *p*≤.001). No differences emerged between SP patients and HCs regarding the extent of their ego-involvement and how unpredictable and challenging the TSST was. Furthermore, in the SP group, the TSST led to a significantly higher increase in state anxiety (STAI-S) as well as a greater decline in good mood (MDBF good versus bad mood; both *p*≤. 01). In addition, restlessness and fatigue increase emerged over time with similar changes in both groups, but SP patients reported more restlessness (MDBF calmness versus restlessness) and fatigue (MDBF tired versus awake) directly before and after the stressor (see again table 2). Consequently, SP patients experienced in contrast to the HCs significantly more negative post-event-processing as measured with the PEPQ (*p*<.001).

**Table 2:** Means (±SD) of self-reported responses to the Trier Social Stress Test (TSST) in patients with social phobia (SP) and healthy controls (HC).

	SP (*n* = 88)		HC (*n* = 78)					Effect
*N* = 166	*M*	(*SD*)	*M*	(*SD*)	Test	*p*	df	size
**MDBF calmness**					Ti: *F* = 44.26	**<.001** ***	1/160	.217
Prä	13.10	(3.69)	17.95	(1.78)	SP: *F* = 150.26	**<.001** ***	1/160	.485
Post	10.78	(4.22)	16.12	(2.77)	TixSP: *F* = 0.63	.428	1/160	
**MDBF awake**					Ti: *F* = 10.36	**.002** **	1/160	.061
Prä	12.95	(3.83)	16.30	(3.13)	SP: *F* = 44.35	**<.001** ***	1/160	.217
Post	12.10	(4.00)	15.54	(3.30)	TixSP: *F* = 0.03	.864	1/160	
**MDBF mood**					Ti: *F* = 75.83	**<.001** ***	1/160	.322
Prä	14.22	(3.14)	18.38	(1.68)	SP: *F* = 183.62	**<.001** ***	1/160	.534
Post	10.94	(3.96)	16.88	(2.26)	TixSP: *F* = 10.51	**.001** **	1/160	.062
**Stai-S**					Ti: *F* = 47.20	**<.001** ***	1/161	.227
Prä	45.49	(9.50)	30.47	(5.18)	SP: *F* = 195.78	**<.001** ***	1/161	.549
Post	52.46	(11.91)	33.59	(7.49)	TixSP: *F* = 6.87	**.01** *	1/161	.041
**PASA**								
Primary appraisal	4.83	(0.83)	3.30	(0.79)	*T* = 12.06	**<.001** ***	163	
Secondary appraisal	3.57	(0.63)	4.63	(0.67)	*T* = −10.40	**<.001** ***	163	
**VAS**								
New	61.28	(30.94)	53.68	(29.29)	*T* = 1.62	.107	164	
Unpredictable	35.09	(30.56)	49.96	(27.48)	*T* = −3.28	**.001** **	164	
Challenge	66.78	(30.40)	67.60	(23.65)	*T* = −0.20	.846	161.36	
Content	22.01	(26.84)	48.04	(27.95)	*T* = −6.12	**<.001** ***	164	
Controllable	33.50	(30.46)	49.15	(29.37)	*T* = −3.36	**.001** **	164	
Threatening	64.90	(32.52)	24.54	(26.73)	*T* = 8.77	**<.001** ***	163.10	
Ego-involvement	67.97	(25.14)	62.81	(24.37)	*T* = 1.34	.183	164	
Stressful	80.93	(21.40)	51.55	(27.78)	*T* = 7.53	**<.001** ***	141.95	
**PEPQ**	40.59	(20.32)	12.90	(11.96)	*T* = 10.73	**<.001** ***	140.05	

*Notes*: *T* =  T-Test, *p* = p-value; df  =  degrees of freedom, *M* =  mean, *S D* =  standard deviation; Ti  =  main effect of time; SP  =  main effect of diagnosis of social phobia; TixSP  =  interaction of time and diagnosis; MDBF  =  multidimensional mental-state questionnaire; Stai-S  =  state version of the State-Trait Anxiety Inventory; PASA  =  Primary Appraisal Secondary Appraisal questionnaire; VAS  =  visual analogue scales; PEPQ  =  Post Event Processing Questionnaire.

* = *p*<.05,

** = *p*<0.01,

*** = *p*<.001.

## Discussion

### Main purpose and highlights of this study

To our knowledge, this study is to date the most comprehensive investigation of stress reactivity, as well as the first to investigate long-term cortisol production and deposition via hair segment analysis in adult SP. Our hypothesis of a generally elevated response to social stress in SP was rejected: SP patients showed no significantly changed physiological (salivary cortisol, plasma cortisol, HR, HRV, sAA, and prolactin) stress response to the TSST – except in the rest phase prior to stress exposure. Here we found elevated cortisol and HR levels in SP patients. Also, the long-term cortisol production and deposition in hair was not altered in SP patients. Nevertheless, the SP patients showed elevated self-reported anxiety before, during, immediately after, and one week after the TSST. This indicates an obvious discordance between self-reported stress experience and physiological stress responses. The pattern of results is highly consistent across the broader range of psychophysiological parameters used in the present study including both short-term and long-term measures. Given that their physiological parameters are overall similar to those of HC's, we would argue SP patients neither show an extreme form of focused fear reactivity (as is typical in phobias) nor of defensive impairment (as is typical for panic disorder or generalized anxiety disorder) (see Lang & McTeague, 2009). Accordingly, our findings confirm the view that SP should be considered “a transition diagnosis on the anxiety spectrum” [50] (p.16).

Our findings of unchanged stress reactivity in SP were obtained in a larger patient sample, and were based on a broader range of observations (i.e., psychophysiological variables) than in any of the previous studies with similar findings [4], [8], [9], [11]–[13]. However, our findings are in contrast to several studies showing significant differences in the acute stress response of SP vs. HC [4], [10], [14]–[20].

This inconsistency between studies may be attributed to non-standardization of stressors and to the investigation of SP in different age groups, as age is known to influence baseline values and/or reactivity to the TSST of several physiological parameters [29]–[31]. Furthermore, participants' responder vs. non-responder status regarding salivary cortisol has to be taken into account: Since non-responders lower the stress response of the whole group, different non-responder-ratios can lead to significantly different stress reactions. Only one other study considered this effect [14]. Finally, as pointed out by Yoon and Joormann [32] the comorbidity status can influence the stress response in SP. Though we found no influence of it in our sample of SP patients, it still might account for the differences reported in literature. Given these methodological problems of studies, which indicated elevated social stress responses in SP, our findings appear to strongly disconfirm the assumption that a more pronounced physiological response to social stress characterizes social phobia.

### SP show anticipatory elevated cortisol and heart rate

While SP patients did not show a significant difference in their physiological stress reactivity, they did present a significant difference in three out of six physiological parameters (salivary cortisol, plasma cortisol, heart rate) during the initial rest phase. Although a number of previous studies in SP have focused on physiological stress reactivity, most of these studies did not find any elevations in anticipation of the stressor [14], [15], [17]. Only two studies reported elevations in physiological response prior to a stressor: Martel et al. [8] found elevated salivary cortisol levels in anticipation of an adapted version of the TSST in adolescent SP girls and HCs – however, there were no significant differences between these groups. The second study by Gerlach et al. [51] found significantly elevated HR in anticipation of an embarrassing situation in SPs compared to HCs.

We cannot fully clarify why most of the studies were unable to document elevations in cortisol and HR in SP patients. The following possible explanations may be taken into account: Firstly, details in the study procedure or the informed consent, which have not been reported in previous studies and are thus not accessible to investigation, could be influential. For example, we explicitly pointed out to the participants that they will partake in a psychosocial stress task. This might have led to anticipatory anxiety and a resulting stress response. Secondly, the experimental situation involving speaking with the investigator and completion of questionnaires is a social situation that SP patients may already view as quite stressful, as opposed to HC. Thirdly, the significant increases in HR and sAA during the rest phase speaks for anticipatory anxiety preceding the TSST since already anticipating an embarrassing situation is associated with elevated HR in SPs compared to HCs [51]. The reason why our results show these changes to be similar between SPs and HCs - as also found by Martel et al. [8] – remains to be investigated. Anticipatory anxiety is a core feature of the clinical picture of SP and, on a neurobiological level, leads to similar activation in the brain in SP patients like a real stressor: Lorberbaum et al. [52] observed that SP patients displayed greater subcortical, limbic, and lateral paralimbic activity (pons, striatum, amygdala/parahippocampus, insula, temporal pole), as well as less cortical activity (dorsal anterior cingulate/prefrontal cortex) under functional magnetic resonance imaging (fMRI) during the anticipation of making public presentations. Along with other structures, e.g., elevated amygdala and reduced prefrontal cortex activity lead to HPA axis activation and sympathetic activation [6]. Moreover, heightened anticipatory anxiety is an important theoretical cornerstone in existing clinical models of SP [53].

In our view, the elevated cortisol and HR levels during the initial rest phase were most likely due to a combination of several factors. In future research, it should be controlled explicitly with assessing subjective anticipatory anxiety during the rest phase several times via appropriate questionnaires.

### The long-term cortisol production and deposition is not changed in SP

Our finding of non-significant differences between SP and HC in a marker of long-term cortisol production, the amount of cortisol incorporated over a three months period into hair, is in line with previous studies. Several investigators likewise found no indication of altered basal cortisol production in SP patients based on endocrine methods covering much shorter time intervals (up to 24 hours in urine, saliva or blood plasma) [26], [27], [54].

### There is an obvious dissociation between self-reported stress experience and physiological stress response in SP

While SP patients exhibited no significant differences in physiological stress parameters in response to the TSST, they reported significantly increased subjective stress – both immediately as well as even one week after the TSST. The results of elevated self-reported anxiety and negative post-event processing are in line with previous studies investigating SP adults and children under acute social stress [4], [9], [10]. This finding is not surprising given that the TSST-situation is predestined to provoke SP symptoms. One possible explanation for this finding is that our SP patients had significantly elevated levels of anxiety sensitivity. Increased levels of anxiety sensitivity have been found already in previous studies in SP patients [55]. Anderson and Hope [55] found that, while adolescent SP did not exhibit higher HR levels compared to HC, they ”were more aware of measured increases in physiological arousal“ (p. 18), and displayed more anxiety to experience physiological arousal which could be seen in their higher ASI-scores. This elevated anxiety sensitivity could be a mediating factor, since it makes SP patients more vulnerable to be worried about their bodily symptoms (even when in the absence of physiological differences compared to HC) and therefore intensifies self-reported anxiety. Moreover, Thibodeau and colleagues recently found that in mostly healthy participants (without a DSM-IV diagnosis) anxiety sensitivity and trait social anxiety were correlated with perceived arousal and state anxiety, but not with objective arousal during three tasks (speech, typing task and hyperventilation) [56].

Finally, as we tested twelve hypotheses (six for the analysis of basal stress level and six for the analysis of stress response) for the physiological parameters and nine for self-reported anxiety and stress, we adjusted the levels of significance using Bonferroni correction to reduce the risk of possible alpha-error inflation. Thus, we derived for the physiological analyses a corrected level of significance of *p_cor_* = .0042 and for the self-reported anxiety and stress a *p_cor_* = .0056. For the biological stress response towards the TSST, all significant findings remained significant after the correction indicating a very robust induction of stress by the TSST. All analyses of self-reported anxiety or stress, except for the MDBF questionnaire, survived alpha-error correction as well. Baseline differences were either not significant beforehand or were no longer significant after correction. Following Beatty and Behnke [57], who reported different stress responses in patients with SP according to the intensity of the stressor applied, these findings still need further investigation.

### Conclusions

In sum, the present findings suggest that SP patients show the same physiological stress response like HCs to a novel psychosocial stressor. Moreover, an apparent discordance between subjective and physiological reactivity to unexpected psychosocial stress for SP patients was documented. Future studies should clarify the role of anticipatory subjective and physiological anxiety responses before social stressful situations. The psychophysiological findings of our study support the more cognitive models of SP [53], [58], which regard biased processing of social information as a central mechanism for the maintenance of this disorder. Our finding underlines the importance to communicate to SP patients that their perceived bodily responses are normal.
